# The Nexus between Workplace Exposure for Wood, Welding, Motor Mechanic, and Oil Refinery Workers and the Prevalence of Prediabetes and Type 2 Diabetes Mellitus

**DOI:** 10.3390/ijerph17113992

**Published:** 2020-06-04

**Authors:** Sultan Ayoub Meo, Thamir Al-khlaiwi, Abdulelah Adnan Abukhalaf, Ali Abdullah Alomar, Omar Mohammed Alessa, Faris Jamal Almutairi, Majed Mohammed Alasbali

**Affiliations:** Department of Physiology, College of Medicine, King Saud University, Riyadh 11461, Saudi Arabia; talkhlaiwi@ksu.edu.sa (T.A.-k.); Abdulelahabukhalaf@gmail.com (A.A.A.); AliAlomarMD@gmail.com (A.A.A.); omar.m.alessa@gmail.com (O.M.A.); Faris11300@gmail.com (F.J.A.); Majedalasbaly@gmail.com (M.M.A.)

**Keywords:** environmental pollution, workplace, prevalence, type-2 diabetes mellitus

## Abstract

Workplace exposure in various occupational and industrial sectors is an emerging health concern worldwide. This study aimed to investigate the nexus between workplace exposure for wood, welding, motor mechanic, and oil refinery workers and the prevalence of prediabetes and type 2 diabetes mellitus. Initially, 2500 male volunteers who were wood, welding, motor mechanic, and oil refinery workers were interviewed. After an examination of their demographics and medical history, 1408 non-smoking wood (158), welding (560), motor mechanic (272), and oil refinery workers (217), along with 201 control subjects, were selected. The participants’ mean age was 36.59 ± 0.29 years and the mean body mass index was 26.14 ± 0.11 kg/m^2^. The selected industry workers had been exposed to their respective wood, welding, motor mechanic, and oil refinery workplaces for 8 h per day, six days per week. The American Diabetic Association (ADA)-based glycated hemoglobin (HbA1c) criterion was used to diagnose prediabetes and type 2 diabetes mellitus. Subjects with an HbA1c of less than 5.7% were regarded as non-diabetics, subjects with an HbA1c of 5.7%–6.4% were considered prediabetics, and subjects with an HbA1c of more than 6.4% were considered diabetics. In wood industry workers, the prevalence of prediabetes (PD) was 64 (40.50%) and in type 2 diabetes mellitus (T2DM), it was 21 (13.29%); in welding workers, the prevalence of prediabetes was 261 (46.60%), and for T2DM, it was 90 (16.07%); in motor mechanic workers, the prevalence of prediabetes was 110 (40.44%), and for T2DM, it was 126 (46.32%); and in oil refinery workers, the prevalence of prediabetes was 80 (36.86%), and for T2DM, it was 35 (16.12%). However; the combined prevalence of prediabetes and T2DM among wood, welding, motor mechanic, and oil refinery workers was 421 (34.79%) and 515 (42.66%), respectively. The prevalence of prediabetes and T2DM among workers increased with the duration of working exposure in the wood, welding, motor mechanic, and oil refinery industries. A one-year working exposure in these industries caused an increase of 0.03% in HbA1c. Workplace exposure in wood, welding, motor mechanic, and oil refinery industries increased the risk of prevalence of prediabetes and T2DM among the workers and affected the diabetes etiology.

## 1. Introduction

Diabetes mellitus is a leading health concern worldwide with a growing occurrence and numerous long-lasting complications. Despite many incredible advancements in medical sciences, diabetes mellitus is still an incurable life-long disease. It is rapidly occurring in all age and gender clusters [1], in both developing and developed nations, and rural and urban regions across the globe. It involves and impairs various physiological functions, organs of the body, and body systems [1], with widely ranging disastrous health problems [2].

The most recent prevalence of diabetes mellitus is 463 million people, and 232 million people are unaware of the fact that they are suffering from the disease. A total of 310 million people with diabetes are living in urban areas, while 153 million people are residing in rural areas. Furthermore, 327 million people with diabetes, i.e., about three-quarters of those with diabetes, are of working age and 366 million diabetic people live in low-income and middle-income countries [3]. Diabetes mellitus currently ranks high on the international health agenda due to it being a major global issue that significantly harms human health and worldwide economies [4]. Many countries across the globe have developed strategies to intervene regarding behavioral risk factors, such as lifestyle, smoking, diet, fast food culture, and physical activity, to reduce the prevalence of diabetes. However, occupational-related environmental pollution has received noticeably less attention from intervention efforts [5].

The global total population is 7.594 billion and the population of labor workers is 3.489 billion people [6]. In 2019, the World Health Organization [7] reported that 70% of workers do not have any insurance for compensation in the case of occupational diseases or injuries. The air pollution in both rural and urban areas has been estimated to cause 4.2 million premature deaths worldwide, mainly in low-income and middle-income countries [7].

Occupational exposure to pollutants generated from industries, including wood [8], welding [9], motor mechanic, and oil refinery [10] industries, is an emerging environmental risk factor that is contributing to the development of acute and chronic diseases. Workers in industrial sectors are exposed to various toxic substances, including fine particulate matter [11]. Particulate matter (PM 2.5 μm) is one of the main pollutants and concerns about it have been increasing because of its damaging impact on human health [12], which is due to its complex composition and wide distribution. The effects of PM on health depend on its size, nature, and chemical composition [13]. Airborne fine particulate matter with an aerodynamic diameter of PM 2.5 μm can penetrate deep into the lungs and gastrointestinal tract (GIT), and pass through the lung and GIT barrier to enter circulating blood. PM 2.5 μm comes from a broad range of chemical and biological sources [13], including nitrogen dioxide, sulfur dioxide, nitrate, ammonium, and organic compounds, as well as polycyclic aromatic hydrocarbons, dust, fumes, and metals [14]. Experimental and epidemiologic studies give support to the concept that work-related exposure to dust and fumes has harmful effects on many biological systems, including the respiratory [15], cardiovascular [16,17], and nervous systems [18].

In recent years, evidence has been accumulating showing that workplace exposure to various occupational and industrial sectors can adversely affect human health [19,20]. However, the literature available right now is mainly based on animal model studies, while human model studies have not been conducted on the workplace exposure in wood, welding, motor, and oil refinery industries and its association with type 2 diabetes mellitus (T2DM). This study aimed to investigate the nexus between workplace exposure in wood, welding, motor mechanic, and oil refinery industries and the prevalence of prediabetes and type 2 diabetes mellitus.

## 2. Subjects and Methods

### 2.1. Study Participants

In this study, the workers from four industries—wood, welding, motor mechanic, and oil refinery—were selected from the Riyadh region, Saudi Arabia. Initially, 2500 male volunteers from these four industries were interviewed, and after demographic and medical history examinations, 1408 non-smoking wood (158), welding (560), motor mechanic (272), and oil refinery (217) workers, along with control subjects (201), were selected. The mean age for the participants was 36.59 ± 0.29 years and the mean body mass index was 26.14 ± 0.11 kg/m^2^. The wood, welding, motor mechanic, and oil refinery workers were exposed to concerning industry pollution for 8 h per day, six days per week. It was ensured that these workers only worked in these four industries and were not exposed to other industries, such as plastic, cement, coal, cotton, or flour factories. The non-smoking control subjects (201) were selected from schools and university clerical staff, technicians, and research assistants. Verbal consent was taken from all the participants who had voluntarily registered to join the research project.

### 2.2. Clinical History and Sociodemographic Characteristics

Three co-investigators interviewed 2500 volunteer male workers and detailed sociodemographic and medical information was obtained. The baseline information, including age, gender, height, weight, BMI, duration of exposure, demographic characteristics, lifestyle, and other health-related information was collected from the questionnaire. Demographic characteristics, including residential address, living conditions, education level, marital status, monthly income, lifestyle information, smoking, and physical activity were recorded. Other health-related evidence, including a family history of diabetes mellitus, was also taken. After demographic, medical history and examination investigations, 1408 non-smoking wood (158), welding (560), motor mechanic (272), and oil refinery (217) workers, along with the control subjects (201), were selected.

### 2.3. Exclusion Criteria

**Figure 1:**
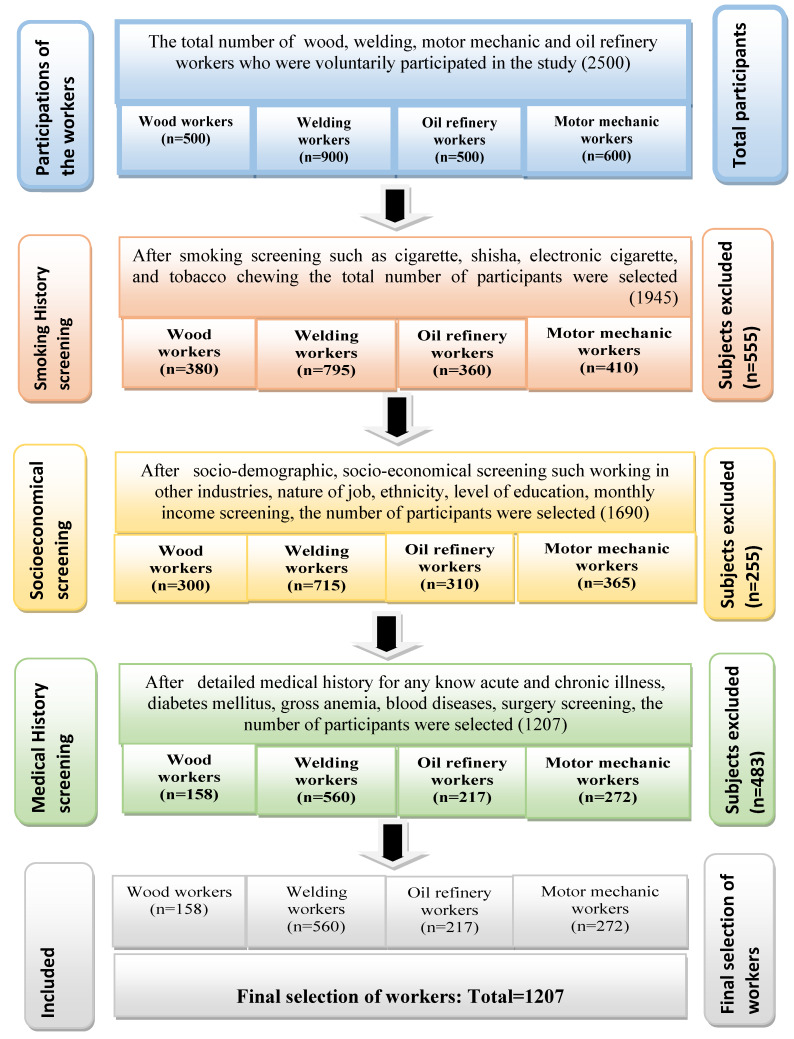
Flow diagram of the selection of wood, welding, motor mechanic, and oil refinery workers.

The participants with a known history of anemia, blood diseases, blood transfusion, asthma, diabetes mellitus, and malignancy were excluded from the study. Subjects with marked obesity were also excluded to minimize the impact of obesity on the prevalence of prediabetes and type 2 diabetes mellitus. The workers who smoked traditional or electronic cigarettes, or shisha, were also excluded [21]. It was ensured that these workers only worked in the wood, welding, motor mechanic, and oil refinery industries. Subjects with a previous history of employment in any other industrial plant that produces dust or fumes, such as plastic, cement, coal, cotton, and flour factories, were also not included in the study [22] (Figure 1).

### 2.4. Measurements of Glycated Hemoglobin (HbA1c)

After a detailed medical interview and gathering demographic data, the selected industry workers were assigned an identification number and a trained paramedic was assigned to measure the HbA1c. The HbA1c was measured using the Clover A1c system (Inforpia, Kyunggi, Korea), which is an automated boronate affinity assay for the determination of the HbA1c percentage in whole blood [23]. The American Diabetic Association (ADA)-based criteria [24] regarding glycated hemoglobin (HbA1c) was used to diagnose diabetes mellitus. Subjects with an HbA1c of less than 5.7% were considered non-diabetics, an HbA1c of 5.7%–6.4% were considered prediabetics, and subjects with an HbA1c of more than 6.4% were considered diabetics [24]. HbA1c is a highly reliable and valid indicator of long-lasting glycemic measurements for the diagnosis of diabetes mellitus [24,25].

### 2.5. Ethics Statement

This study was executed in harmony with the “Declaration of Helsinki,” and the protocol was approved by the Institutional Review Board, Ethics Committee, College of Medicine Research Centre, King Saud University (E-18-3654).

### 2.6. Statistical Analysis

The data were entered into the computer, where the IBM SPSS Version 22 and Microsoft Windows software, Armonk, NY, USA were used. Continuous variables were expressed as the mean ± standard deviation and descriptive data were expressed as frequency (%). The frequencies and percentages for the prevalence of prediabetes and type 2 diabetes mellitus, their association with sociodemographic data, and the duration of exposure were calculated using chi-square tests of independence. To identify the independent risk factor for HBA1c, a multiple linear regression model was used. The level of significance was taken to be *p* < 0.05.

## 3. Results

The anthropometric features of the wood, welding, motor mechanic, and oil refinery workers, as well as the control group, are presented in Table 1. The mean age for the participants was 36.59 ± 0.29 years and the mean body mass index was 26.14 ± 0.11 kg/m^2^. These workers were exposed in the wood, welding, motor mechanic, and oil refinery industries for 8 h daily, six days a week. The mean duration of exposure to these industries was 8.41 years (Table 1).

In the wood industry workers, the prevalence of prediabetes was 64 (40.50%) and for T2DM, it was 21 (13.29%); in welding workers, the prevalence of prediabetes was 261 (46.60%) and for T2DM, it was 90 (16.07%); in motor mechanic workers, the prevalence of prediabetes was 110 (40.44%) and for T2DM, it was 126 (46.32%); and among oil refinery workers, the prevalence of prediabetes was 80 (36.86%) and for T2DM, it was 35 (16.12%) (Table 2). Among the workers, the association of prediabetes and T2DM was linked with the working duration in these industries (Table 3). However, there was no relationship identified between BMI and the prevalence of prediabetes or T2DM among the industry workers (Table 3).

The combined prevalence of prediabetes and T2DM among wood, welding, motor mechanic, and oil refinery workers was 420 (34.79%) and 515 (42.66%), respectively (Table 2). The prevalence of prediabetes and T2DM among these workers was significantly increased with the duration of working exposure in these four industries. After adjustment for age and BMI, and based on the findings of a multiple regression analysis for HBA1c between the control and exposed subjects, it was found that the HBA1c was significantly higher among welding, motor mechanic, oil workers compared to the control group (Table 2). The one-year working exposure to these workers caused an increase of 0.03% in HbA1c (Table 3). It was also found that HBA1c was significantly higher among the workers in these four industries compared to BMI-matched control subjects (Table 3).

## 4. Discussion

To the best of our knowledge, this is the first study added in the medical literature that investigated the nexus between workplace exposure to the wood, welding, motor mechanic, and oil refinery industries and the prevalence of prediabetes and type 2 diabetes mellitus. In this study, a positive correlation was identified between the prevalence of prediabetes and T2DM and exposures to the wood, welding, motor mechanic, and oil refinery industries.

At the end of the 20th century, the overall understanding regarding the occurrence of type 2 diabetes mellitus was that it was due to eating unhealthy food, a lack of exercise, and genetics; however, in recent years, environmental pollution is becoming known as an emerging causative mechanism in the development of T2DM [26].

Paul et al. [27] found that air pollutants, especially nitrogen dioxide (NO_2_), was linked to an increased risk of diabetes mellitus. As NO_2_ is frequently found in and near road traffic, this may indicate that traffic-related air pollution has a strong impact on diabetes etiology. In another study, Liu et al. [28] found that a higher residential exposure of air pollutant concentrations of PM 2.5 μm and NO_2_ increased the odds of developing T2DM and high fasting blood glucose levels.

Similarly, Meo et al. [20] reported that the prevalence of prediabetes and T2DM among plastic industry workers was increased with the duration of working exposure in the plastic industry. In another study, Meo et al. [22] also found that cement industry pollution is linked with a prevalence of prediabetes and T2DM. Meo et al. [19] demonstrated that environmental pollution is a major cause of insulin resistance and the incidence of type 2 diabetes mellitus. Correspondingly, in the present study, the prevalence of prediabetes and T2DM was identified in wood, welding, motor mechanic, and oil refinery workers, which was associated with the duration of working exposure in these factories. However, no association was established between BMI and the prevalence of prediabetes and T2DM among these industry workers (Table 3).

Kelsall et al. [29] found similar findings, where they investigated the association between the risk of T2DM with various occupational and industrial working populations. Increased diabetes risks were found in various occupational groups who worked in industries compared to the office workers. The authors concluded that the blue-collar industry workers had a high diabetes risk. Similarly, Yang et al. [30] also reported a high correlation between metal workers and the risk of prediabetes and diabetes mellitus. In animal-model-based studies, Wang et al. [31], Balti et al. [32], Park and Wang [33], Eze et al. [34], and Bellou et al. [35] reported that prolonged exposure to higher concentrations of environmental air pollutants is significantly associated with an increased risk of type 2 diabetes mellitus. In the present study, we identified that exposure to wood, welding, motor mechanic, and oil refinery industries is associated with a high prevalence of prediabetes and T2DM.

### 4.1. Pollutant Mechanisms That Cause Insulin Resistance and T2DM

**Figure 2:**
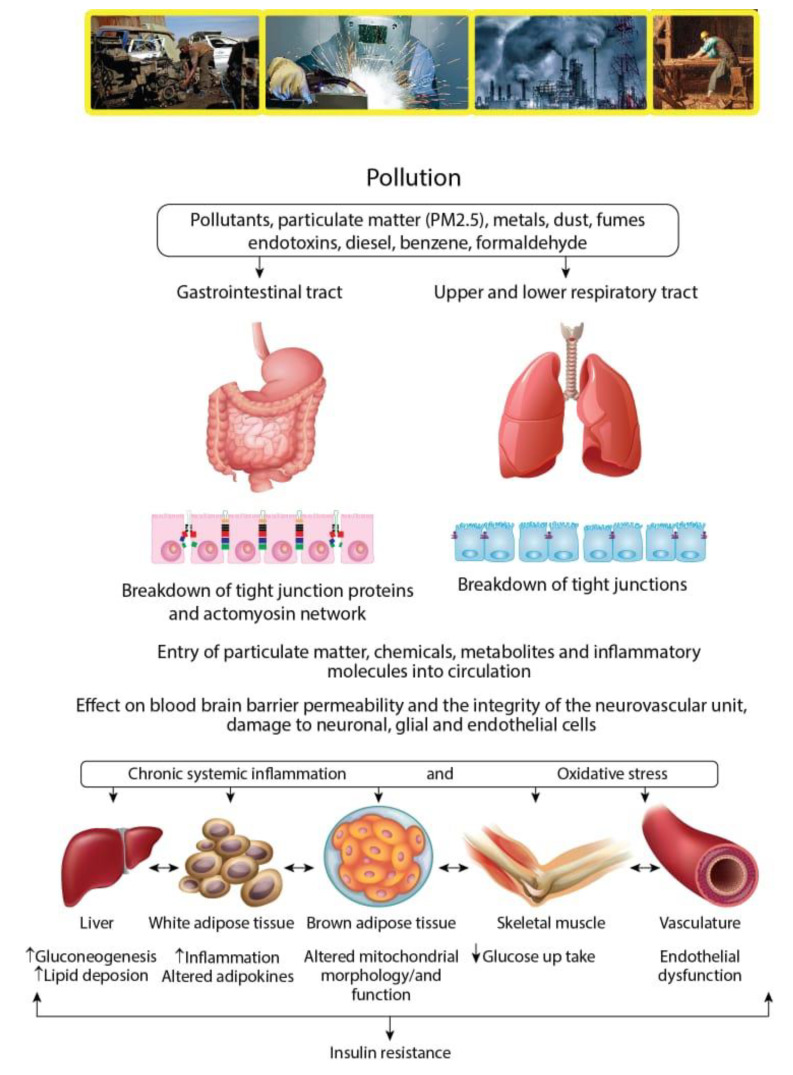
Mechanisms taking place during work exposure to pollutants and their entry into the human body that cause insulin resistance. UFPM: Ultrafine Particulate Matter.

The epidemiologic and experimental literature acknowledges that environmental pollutants enhance the risk of insulin resistance and ultimately leads to type 2 diabetes mellitus (Figure 2). Air pollutants may contribute low-grade inflammation and oxidative stress [36,37,38], pancreatic disruptions [39], decreased insulin signaling, glucose metabolism impairment, insulin resistance, and type 2 diabetes mellitus [37,38] (Figure 2). These are the possible mechanisms allowing pollution to cause insulin resistance and the prevalence of prediabetes and T2DM.

### 4.2. Study Strengths and Limitations

Similar to other studies, this study has strengths and limitations. This is the first study to investigate the prevalence of prediabetes and T2DM among multi-industrial workers, including wood, welding, motor mechanic, and oil refinery industrial workers. The study exclusion criteria were highly standardized and cigarette smokers were excluded. The American Diabetes Association diagnosis approach was employed, which was based on HbA1c levels and all the participants were categorized as either normal, prediabetics, or diabetics. The glycated hemoglobin (HbA1c) is a more reliable and valid indicator for identifying an individual’s long-term mean blood glucose levels. Therefore, this work could be considered a best reference regarding the industrial allied pollution and prevalence of prediabetes and T2DM since in this study, four different industries (wood, welding, motor mechanic, and oil refinery) were investigated.

However, the limitations in the present study are as follows: First, despite trying to recruit many workers from the wood, welding, motor mechanic, and oil refinery industries, the majority of employees were cigarette smokers; therefore, we excluded several workers and finally included 1207 workers. Second, we were unable to control for some potential confounders, such as indoor air pollution exposures, due to the unavailability of this information. The omission of these potential confounders may affect the extent that pollution has when potentially causing diabetes.

## 5. Conclusions

Workplace exposure in the wood, welding, motor mechanic, and oil refinery industries is associated with a high prevalence of prediabetes and T2DM. The prevalence was associated with the duration of working exposure in these occupational settings. The results indicate that wood, welding, motor mechanic, and oil refinery pollution affects diabetes etiology. The industry workers should wear respiratory protective equipment to minimize the risk of dust, fumes, and pollutant exposure. The working conditions have a powerful impact on health equity, and environmental health officials could provide important interventions for protecting workers’ health, both in large- and small-scale industries to improve working conditions and provide early detection of occupationally caused prediabetes and T2DM among the workers. The present study’s findings have significant public health implications as the findings support the extension of diabetes intervention efforts to include environmental risk factors that may contribute to diabetes etiology. Future prospective studies with broader geographic areas are still needed to further verify the present study’s results and to confirm the correlation between work-produced pollutants and T2DM.

## Figures and Tables

**Table 1:** Sociodemographic and clinical characteristics of wood, welding, motor mechanic, and oil refinery workers, as well as the control group (*n* = 1408).

Variables	Wood Workers (*n* = 158)	Welding Workers (*n* = 560)	Motor Mechanic Workers (*n* = 272)	Oil Refinery Workers (*n* = 217)	Control Group (*n* = 201)
Age (years)	41.23 ± 9.76	35.41 ± 9.49	35.67 ± 10.46	37.0 ± 10.64	31.38 ± 6.40
BMI (kg/m2)	25.30 ± 3.14	25.45 ± 3.45	26.57 ± 4.53	27.34 ± 4.71	24.88 ± 2.36
Exposure (years)	10.47 ± 0.87	6.68 ± 6.56	14.42 ± 8.98	1.00 ± 0.80	-
HbA1c (%)	5.70 ± 0.86	5.98 ± 0.98	7.25 ± 2.02	6.09 ± 1.42	5.39 ± 0.46

Values are expressed in mean ± SD.

**Table 2:** Prevalence of prediabetes and type 2 diabetes mellitus among wood, welding, motor mechanic, and oil refinery workers (*n* = 1207).

Parameters	Prevalence of Prediabetes and Type 2 Diabetes Mellitus				
	Wood Workers (*n* = 158)	Welding Workers (*n* = 560)	Motor Mechanic Workers (*n* = 272)	Oil Refinery Workers (*n* = 217)	Combined Prevalence (*n* = 1207)
Normal: HbA1c < 5.7%	73 (46.20%)	209 (37.23%)	36 (13.23%)	102 (47.00%)	420 (34.79%)
Prediabetes: HbA1c 5.7%–6.4%	64 (40.50%)	261 (46.60%)	110 (40.44%)	80 (36.86%)	515 (42.66%)
Diabetes: HbA1c > 6.4%	21 (13.29%)	90 (16.07%)	126 (46.32%)	35(16.12%)	272 (22.53%)

Values are expressed as a frequency (percentage). The American Diabetes Association criteria for HbA1c was used to classify prediabetes and diabetes mellitus [24].

**Table 3:** Multiple regression analysis for HbA1c between subjects exposed in wood, welding, motor mechanic, and oil refinery industries, as well as the control group (*n* = 1408).

		HbA1c				
Covariate	Level	B	95% CI Low	95% CI High	Type B *p*-Value	Type 3 *p*-Value
Group	Motor mechanic	1.71	1.48	1.93	<0.001	<0.001
	Oil	0.47	0.23	0.71	<0.001	
	Welding	0.46	0.27	0.66	<0.001	
	Wood	0.20	−0.06	0.46	0.138	
	Control	-	-	-	-	
Duration	One-year increase	0.03	0.02	0.04	<0.001	<0.001
BMI	One-unit increase	0.01	−0.01	0.03	0.264	0.264

B—Regression coefficient; type B *p*-value: overall association; type 3 *p*-value: overall association.
